# SynBio and the Boundaries between Functional and Pathogenic RepA-WH1 Bacterial Amyloids

**DOI:** 10.1128/mSystems.00553-20

**Published:** 2020-06-30

**Authors:** Rafael Giraldo

**Affiliations:** a Department of Microbial Biotechnology, National Center of Biotechnology (CNB-CSIC), Madrid, Spain; UiT—The Arctic University of Norway

**Keywords:** RepA-WH1, bacterial amyloids, prions, protein engineering, synthetic biology

## Abstract

Amyloids are protein polymers that were initially linked to human diseases. Across the whole Tree of Life, many disease-unrelated proteins are now emerging for which amyloids represent distinct functional states. Most bacterial amyloids described are extracellular, contributing to biofilm formation. However, only a few have been found in the bacterial cytosol. This paper reviews from the perspective of synthetic biology (SynBio) our understanding of the subtle line that separates functional from pathogenic and transmissible amyloids (prions). In particular, it is focused on RepA-WH1, a functional albeit unconventional natural amyloidogenic protein domain that participates in controlling DNA replication of bacterial plasmids. SynBio approaches, including protein engineering and the design of allosteric effectors such as diverse ligands and an optogenetic module, have enabled the generation in RepA-WH1 of an intracellular cytotoxic prion-like agent in bacteria. The synthetic RepA-WH1 prion has the potential to develop into novel antimicrobials.

## Abstract (precis)

Amyloids are protein polymers that were initially linked to human diseases. Across the whole Tree of Life, many disease-unrelated proteins are now emerging for which amyloids represent distinct functional states. Most bacterial amyloids described are extracellular, contributing to biofilm formation. However, only a few have been found in the bacterial cytosol. This paper reviews from the perspective of synthetic biology (SynBio) our understanding of the subtle line that separates functional from pathogenic and transmissible amyloids (prions).

## INTRODUCTION

Amyloids are protein β-sheet aggregates made of an indefinite number of subunits inserted through sequence segments with the potential to form β-strands. Amyloids can adopt various aggregated forms, most commonly fibrillar and oligomeric species, through which proteins lose their natural function to gain a cytotoxic phenotype (reviewed in reference 1). Amyloid aggregates are known for their involvement in many fatal human neurodegenerative and systemic diseases. The mammalian prion protein (PrP) is a particular type of amyloid that can be “horizontally” transmitted between organisms as the causal infectious agent of spongiform encephalopathies, but recently this property has also been attributed to the intraorganismic cell-to-cell transmission of many other amyloids involved in neurodegeneration (e.g., in Alzheimer’s, Parkinson’s, and Huntington’s diseases and amyotrophic lateral sclerosis), which thus are considered prion-like proteins (also known as prionoids) (reviewed in reference 2).

However, “vertically” inheritable (from mother to daughter cells with cell division) aggregated forms of proteins, often transcription or translation factors, have also been characterized as prions in yeast. Upon amyloid aggregation, usually involving Gln/Asn-rich domains, yeast prions readily switch their normal function off, thus leading to the expression of alternative physiological programs. These switches, in response to environmental and metabolic challenges, confer a selective advantage to a subpopulation of the cells, which can be epigenetically inherited with the aggregates to the progeny during many generations. Functional amyloids were later found in metazoan and plant cells, associated with phenotypes such as skin pigmentation, hormone storage, long-term synaptic potentiation (memory imprinting), or vernalization (plant adaption to prolonged cold) (reviewed in references 3 to 5). Functional amyloids and prion-like proteins have also been described in bacteria.

## BACTERIAL AMYLOIDS HAVE BOTH BRIGHT AND DARK SIDES

Since the discovery of the amyloid nature of the curli (CsgA) protein fibers that form part of Escherichia coli biofilms (6), it has been found that analogous extracellular amyloids are built by other species in the *Bacteria* life domain (reviewed in references 7 and 8). Coupled to protein synthesis at the cytosol, CsgA is transferred to the periplasm and subsequently exported to the extracellular space with the help of cofactors encoded in the *curli* operon. CsgA becomes folded upon fibrillar polymerization on the surface of the bacterial cell (reviewed in reference 9). This trail has been traced with molecular detail for the genesis of curli and other extracellular amyloids in Gram-negative bacteria, in particular *Pseudomonas* FapC (10). In Gram-positive bacteria, the paradigmatic functional extracellular amyloid is Bacillus subtilis TasA (11, 12), which accommodates its secretion pathway to the lack of an external membrane. *Staphylococcus* assembles various functional amyloids such as Bap (13), or the phenol-soluble modulins PSMα1 and PSMα4, which form cross-β amyloid fibers scaffolding the extracellular matrix, while PSMα3 forms amyloid-like fibers, but made of stacked α-helices (14). These proteins have probably evolved to readily assemble as amyloid fibers, conferring a selective advantage to bacteria by scaffolding an extracellular matrix with a net adaptive value in colonizing environmental niches. With a completely different function, microcin (Mcc) E492, secreted by *Klebsiella* cells, can assemble as an extracellular amyloid that neutralizes the antibacterial activity of the protein (15).

**FIG 1:**
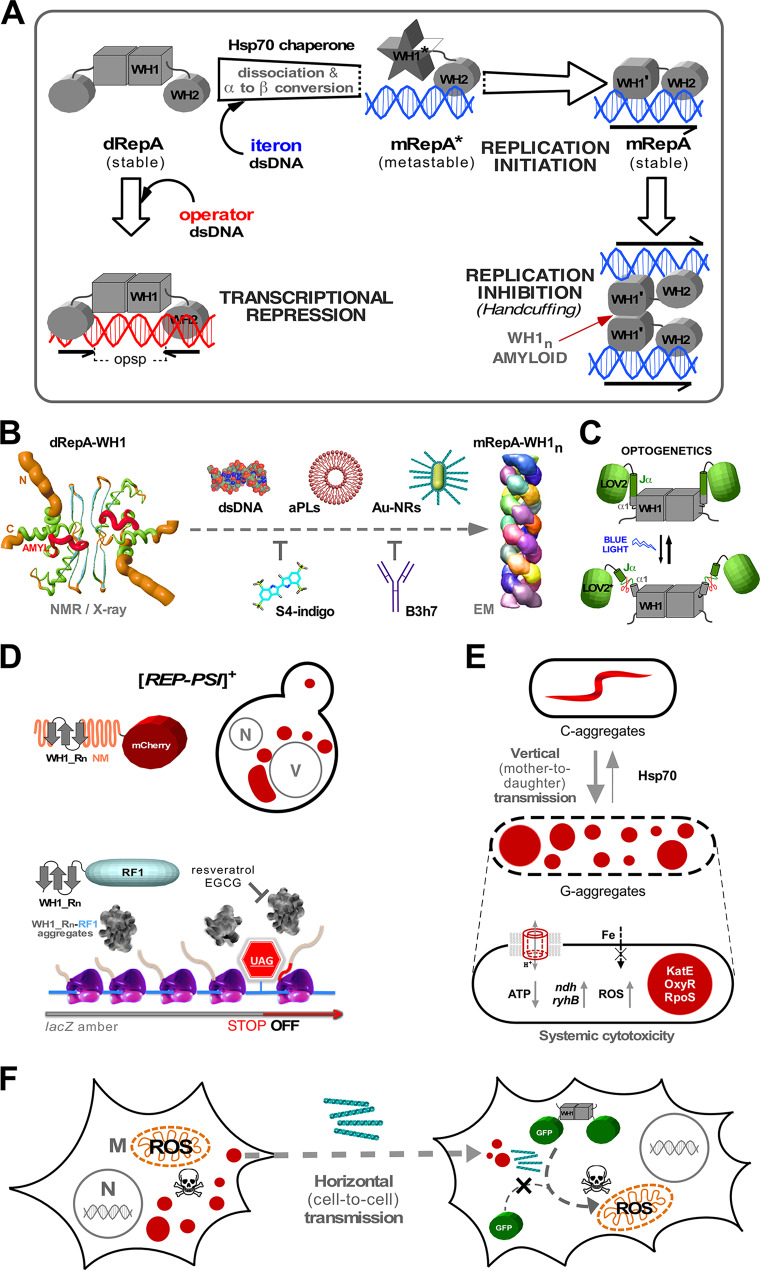
The bacterial functional amyloid RepA as a “generic” model of amyloidosis. (A) In many bacterial plasmids, RepA is a dimeric transcriptional self-repressor that, on binding to DNA direct repeats, dissociates as monomers to initiate replication. Then, its WH1 domain “handcuffs” replicated plasmids together by assembling an amyloid oligomer that hinders premature reinitiation. (B) Fraying N- and C-terminal helices (orange) in RepA-WH1 dimers (left) prime RepA-WH1 dissociation and the assembly of the monomers as helical filaments (right), involving an amyloidogenic loop (L_26_VLCAASLI_34_, red). Amyloidogenesis can be driven (middle) by natural allosteric ligands such as DNA and vesicles including acidic phospholipids (aPLs), in which RepA-WH1 forms pores, or by gold nanoparticles (Au-NRs) functionalized with the protein. Amyloid assembly can be counteracted *in vitro* by indigotetrasulfonate (S4-indigo), which competes with DNA binding and locks dimers, or by an antibody (B3h7) targeting the amyloidogenic conformation. (C) Fusion of a plant photosensor domain (LOV2) to the N-terminal helix in RepA-WH1 enables optogenetic modulation of amyloidogenesis: blue light illumination results in unfolding of the chimeric Jα-α1 helix, generating cytotoxic amyloid oligomers. (D) Repeats of the amyloidogenic stretch in RepA-WH1 (Rn) can either functionally replace prionogenic NM sequences in the yeast prion [*PSI*^+^], forming perivacuolar (V) rather than perinuclear (N) aggregates (top). The same repeats fused to the E. coli releasing factor RF1 enable stop codon read-through by ribosomes, which is reverted by antiamyloid compounds (resveratrol, epigallocathequin-3-gallate [EGCG]) (bottom). (E) RepA-WH1 fused to fluorescent proteins (red) is vertically inherited in E. coli as either of two distinct amyloid strains: one forms multiple globular foci (G), blocking division and eliciting cell death, while another forms single comet-shaped (C) harmless particles. An Hsp70 chaperone detoxifies aggregates by favoring the C strain (top). RepA-WH1 oligomers make pores at the inner membrane, thus decreasing ATP synthesis and iron transport, which induces expression of the NdhII dehydrogenase that enhances the production of H_2_O_2_. Coaggregation with RepA-WH1 of key proteins dismantles the responses to membrane and oxidative stresses (bottom). (F) Horizontal spread of RepA-WH1, either as aggregates expressed in donor cells or as fibers assembled *in vitro*, can be achieved in mammalian cells. Cytotoxicity in the recipient cells is conditional to the heterologous expression of malleable RepA-WH1 and has mitochondria (M), organelles of bacterial ancestry, as the main target. In summary, RepA-WH1 is a synthetic bacterial prion-like protein that, although unrelated to the human proteome, illuminates a minimal core of events leading to an amyloid disease, and whose toxicity in bacteria can be tuned on purpose.

Many bacterial proteins with an intracellular location can be experimentally assembled as amyloid fibers *in vitro*. However, intracellular functional bacterial amyloids are scarce compared to the extracellular ones. RepA, a manifold protein from the plasmid pPS10 of the phytopathogen Pseudomonas savastanoi (Fig. 1A), assembles as very stable dimers, a form in which it is a transcriptional self-repressor. When dimers dissociate, the resulting metastable monomers bind to the plasmid origin of replication at directly repeated sequences (iterons) to initiate DNA replication (16). The switch between these alternative functions is modulated at the level of the RepA N-terminal domain (WH1), which mediates dimerization but can experience a conformational change that uncovers a DNA binding activity, accessory to the main DNA recognition determinant (WH2) (16). Finally, the origin-bound monomers, which are aggregation-prone, while still bound to the iterons can link together (“handcuff”) two plasmid copies to inhibit premature replication rounds (17). WH1 in handcuffed RepA forms an amyloid oligomer, constituting an early case for a functional intracellular bacterial amyloid, and also for an amyloid controlling DNA replication (18). A second example of an intracellular functional amyloid is the transcriptional terminator CbRho from Clostridium botulinum, whose aggregation relies on a unique N-terminal Asn-rich domain with similarities to the amyloidogenic domains in yeast prions (19). CbRho aggregation enables RNA polymerase to overcome termination of transcription. This alters the bacterial transcriptome, while vertical transmission of the aggregates determines a way of cytoplasmic inheritance of the acquired phenotypes (20). Other regulatory RNA binding proteins, such as E. coli Hfq (21), also use amyloidogenic domains to control their functional assembly.

## WHEN THE DEVIL IS IN THE DETAIL: HOW BACTERIA KEEP THE BEAST UNDER CONTROL

Amyloids can be generated either by intrinsically disordered proteins (IDPs) or by folded protein domains, showing a net dependence on sequence composition (22). Attaining a thermodynamically stable protein globular fold depends on hydrophobic cores that usually are made of aggregation-prone stretches, so that both are evolutionarily coselected. Amyloidogenicity is then softened through strategically positioned hydrophilic “gatekeeper” residues (23). Amyloid fiber growth follows a nucleated polymerization sigmoidal kinetics in which the generation of thermodynamically unfavorable (slow) amyloid nuclei is followed by favored (fast) addition of monomers to the nascent fiber ends (1). Kinetics plays another key role: while in functional amyloids quick-folding trajectories of assembly reduce the chance to get trapped into risky aggregation-prone local free energy minima, in pathogenic amyloids their slower folding/assembly kinetics leave plenty of time to fall into any such energy wells, leading to unproductive cytotoxic aggregation (1, 9). So, it is common that functional amyloids are enriched in dynamic IDPs, often as repeats with low sequence complexity (e.g., yeast prion domains), albeit such regions can also be found in pathogenic amyloids (Tau, α-synuclein, TDP-43) (22). IDPs are regulatory interfaces of liquid-liquid phase separations in proteins with a central role in the normal physiology of cells that, when subverted to hydrogel/amyloid states, result in disease (reviewed in references 24 and 25). In proteins with stable native folds (e.g., β2-microglobulin, superoxide dismutase, lysozyme, transthyretin, p53), pathogenic amyloidogenicity often is the result of destabilization through mutation, subunit dissociation, proline isomerization, or posttranslational modifications (such as proteolysis or age-correlated oxidation) (1, 26). A hallmark of pathogenic amyloids is structural polymorphism in their cross-β fibers, which is linked to the propagation and characteristic phenotypes (cytotoxicity and course of disease) of distinct prion-like strains (1, 27). In contrast, functional amyloids tend to acquire unique (i.e., nonpolymorphic) well-defined three-dimensional (3D) structures upon assembly, quite often β-helices or solenoids (9), albeit exceptions exist, such as natural truncated peptides from staphylococcal PSMα3, which form polymorphic cross-β structures, distinct from the cross-α fibers characteristic of the full-length protein, while gaining a toxic activity against other bacteria (28).

Curli extracellular amyloids are built from intrinsically disordered CsgA monomers, which get quickly folded *in vitro* upon their addition at the growing end of an amyloid fiber (29), although whether this is also the case *in vivo* is currently unknown. CsgA carries five repeats of an amyloidogenic stretch adopting a strand-loop-strand fold, of which the two at the edges, in neighbor subunits within a fiber, mutually recognize in a head-to-tail orientation to mediate CsgA polymerization, while the three central repeats slow down the kinetics of assembly. The initial nucleation of fiber growth is made by the CsgB, which includes four repeats that are sequence related to those in CsgA, but the lack of the fifth one avoids self-assembly (30). The location of these proteins at the bacterial extracellular surface enables curli amyloid formation to be regulated by compartmentalization (9). Upon its cytoplasmic synthesis, export of CsgA to the periplasm, through the general inner membrane channel SecY/E/G (31), is followed by binding to specialized chaperones (CsgC/H) that avoid CsgA misfolding in this intermediate compartment, as indicated by the fact that the deletion of *csgC* leads to cell death (32). CsgA then crosses the outer membrane through an exclusive oligomeric channel (CsgG) with the assistance of auxiliary proteins (CsgE/F) (33). Extensive work on the *Pseudomonas* Fap system has revealed common features to those found in curli, in terms of both amyloid structure and kinetics of nucleated polymerization, with FapC as the protein constituting the fibers on three amyloidogenic repeats, FapB as the nucleating factor, FapA playing the role of periplasmic chaperone, and FapF as the export channel (10, 34). In summary, bacteria deploy a complex vectorial transport pathway to efficiently exclude the intracellular accumulation of potentially toxic misfolded intermediates of proteins that, when normally displayed at the cell surface, have net selective value in biofilm scaffolding. Interestingly, bacteria expressing curli can cross-seed the aggregation of proteins involved in neurodegenerative diseases (35), such as α-synuclein when overexpressed in worms or in mice (36, 37), pointing to a possible role for amyloids from gut microbiota in triggering neuroinflammation.

Unlike in curli-related amyloids, posttranslational covalent modification of MccE492 with salmochelin, a catechol-siderophore molecule, enables secretion of the peptide in a soluble, cytotoxic form competent to bind to the siderophore receptors on the surface of target cells (38). MccE492 modification occurs in response to low levels of iron, thus enabling microcin secretion as a competitive adaptation of bacteria in a scenario of environmental shortage of an essential resource (39). While unmodified MccE492 can assemble extracellularly as amyloid fibers that inactivate its toxicity, mutations enhancing amyloidogenicity can lead to the intracellular accumulation of protein aggregates (15).

RepA protein modulates the assembly of functional amyloids to inhibit plasmid replication through a conformational change triggered by specific, allosteric binding to each one of four 22-bp DNA sequence repeats (iterons) found at the plasmid origin of replication (Fig. 1A) (16). Recent findings reveal that transient, low-affinity DNA binding to the RepA-WH1 domain would destabilize its β-sheet dimerization interface by breaking the network of cation-π interactions between amino acid side chains that contribute to hold together the two protein subunits, thus increasing the intrinsic instability of the N- and C-terminal ends in WH1 (Fig. 1B, left) (40). Such WH1 destabilization unleashes an internal, highly flexible amyloidogenic loop to initiate self-assembly with a mirrored RepA molecule bound in *trans* to a second plasmid molecule, into a complex that blocks reinitiation (17, 18). While transient binding to one end of an iteron DNA repeat triggers WH1 amyloidogenesis, recognition with higher affinity of the other half of each iteron by WH2, the C-terminal domain in RepA, limits the polymerization of the protein on the DNA template to the extension spanned by the iterons (17, 41). Thus, sequence-restricted DNA binding is the way in which RepA achieves a defined structure as a functional amyloid, avoiding indefinite polymerization that could lead to cytotoxic amyloid aggregation.

It is noteworthy that functional RepA amyloids are built on a very stable domain (WH1) that, to be remodeled for amyloid assembly, requires binding of a natural allosteric ligand (42). This is similar to amyloidogenesis of the mammalian prion PrP and p53 that can be promoted by binding to nucleic acids (43). Remarkably, densities attributable to unidentified small molecules have been recently found in the cryo-electron microscopy (cryo-EM) structures of amyloid fibers in pathogenic (44) and functional (45) amyloids. Somehow, the dependency of RepA assembly on ligand binding resembles the polymerization of natural cytoskeletal fibers (i.e., actin, tubulin), in which nucleotide binding and hydrolysis play the role described here for DNA. However, the assembly of the cytoskeleton is a reversible dynamic process, whereas amyloid assembly is irreversible, being counteracted by protein chaperones and/or dismantled by the proteasome or by autophagy (46). In the case of RepA, the cytosolic Hsp70 chaperone in Gram-negative bacteria (DnaK) provides a second level of regulation by modulating the conformational dynamics of WH1 (47, 48).

## HOW TO ACHIEVE CONTROL ON AMYLOID CONVERSION THROUGH SynBio

Synthetic biology (SynBio) seeks the implementation of novel functionalities in biological systems, at any level of organization, by using either preexistent natural or new artificial constructive resources (49). The potential of amyloid assemblies in SynBio as constructive nanomaterials is just exploding (50–52). Bacterial extracellular amyloids pioneered the implementation in biofilms of novel bioengineering solutions by exploiting the ability of heterologous C-terminal fusions to CsgA to retain their competence for secretion and assembly. In this way, distinct peptide tags have empowered curli amyloid fibers with metal coordination, nanoparticle templating, covalent protein immobilization, or affinity binding properties *in vitro* (53). The resulting bacterial biofilms exhibit self-regenerating hydrogel architectures (54) or electric conductivity (55) or can be used to program mouse microbiota to counteract inflammatory bowel disease (56). In these approaches, bacterial extracellular amyloids meet the assembly selectivity required for orthogonal β-strand functional scaffolding (57).

In the case of the RepA functional intracellular amyloids (see above), much effort has been put into finding through SynBio alternative ways to control amyloidogenesis (Fig. 1B, center). The first approach was testing mutant variants of RepA-WH1 as parts with improved properties. Among the few mutations found in genetic screenings to modulate the activity of RepA in replication, the most frequent one was Ala31 changed to Val (A31V), located in the amyloidogenic stretch in WH1 (17). *In silico* predictions and *in vitro* assays showed that A31V substantially enhances the assembly of RepA-WH1 into amyloid fibers (42), formed by the lateral association of several thin (4-nm-wide) filaments, in which protein monomers polymerize as single or double helical threads with a tubular section (Fig. 1B, right) (41).

Besides double-stranded DNA, other ligands have been tested or developed as modulators of RepA-WH1 amyloidogenesis *in vitro* (Fig. 1B, above arrow). Among them, it is remarkable that lipid vesicles including acidic phospholipids (cardiolipin, phosphatidylglycerol) promote both the aggregation of RepA-WH1 and its insertion in such model membranes, forming annular oligomeric pores with a diameter similar to that found in the amyloid fibrillar tubules (58). Display on membranes of negatively charged phospholipid heads may provide a two-dimensional pattern recognized by RepA-WH1, analogously to phosphates in the backbone of effector DNA (42). Membrane insertion coupled to the formation of pores is a phenomenon common to many proteins involved in human amyloidoses (59). Exploring surface effects on amyloidogenesis, RepA-WH1 functionalized on gold nanorod particles forms (heat and detergent) denaturation-resistant oligomers that seed the growth of amyloid fibers, generating a signature in surface-enhanced Raman scattering (SERS) spectrum that can be used to monitor amyloid polymerization (60).

Regarding the possibility of generating specific inhibitors of amyloidogenesis (Fig. 1B, below arrow), indigotetrasulfonate (S4-indigo) was identified through virtual screening as a ligand of an Arg-rich patch in RepA-WH1 that is involved in DNA binding. *In vitro* assays showed that this molecule indeed binds to the protein with submicromolar affinity and it is able to block the assembly of RepA-WH1 fibers as an allosteric inhibitor (61). Recent nuclear magnetic resonance (NMR) studies reveal that S4-indigo also prevents amyloidogenesis through a second mechanism: binding to a pocket between the unstable N-terminal α-helix and a β-hairpin involved in dimerization, thus blocking DNA-promoted dissociation of RepA-WH1 dimers (40). Another tool generated to get control on RepA-WH1 amyloidogenesis was B3h7, a monoclonal antibody specific for the metastable conformation of RepA-WH1 that recognized oligomers on the pathway leading to amyloid fibers, thus inhibiting their assembly (62). B3h7 was instrumental in identifying the nucleoid as the place for the initial assembly of RepA-WH1 amyloids (62), and the handcuffed iteron-bound RepA complexes as amyloid oligomers (see above) (18).

On top of those developments, the optogenetic fusion of a plant, blue-light-responsive, phototropin LOV2 domain to RepA-WH1 has recently enabled, in a physical way, riding the folding landscape of this bacterial protein toward amyloidogenesis (Fig. 1C) (63). In the LOV2-WH1 chimera, the C-terminal helix in LOV2 (Jα) is fused in frame to the N-terminal, unfolding-prone (see above) (40) helix (α1) in RepA-WH1. The absorption of blue light photons by the flavin mononucleotide (FMN) cofactor in the plant domain unfolds Jα, and thus also the fused α1, which transmits its destabilization to the core of WH1. When used to seed amyloidogenesis *in vitro*, under blue light illumination the LOV2-WH1 chimera templates the assembly of RepA-WH1 oligomers, while in darkness the protein leads to fiber bundles and extended sheets (63). At the macroscopic level, switching blue light off results in an irreversible liquid-to-hydrogel phase transition in LOV2-WH1 when tagged with a fluorescent protein (63).

The amyloid-prone hydrophobic stretch in RepA-WH1(A31V) (L_26_VLCAVSLI_34_) is by itself a suitable part for the development of synthetic devices when engineered into known functional amyloids. This was demonstrated by replacing the oligopeptide Q/N-rich polar repeats in the yeast protein Sup35 by up to five copies in tandem of the amyloidogenic RepA-WH1 peptide, thus generating a series of novel hybrid prions, [*REP-PSI*^+^], that mimic the phenotype of the natural yeast prion [*PSI*^+^] (3): reading through stop codons in translation (Fig. 1D, top) (64). This constructive design was then applied to fuse the same RepA-WH1 repeats to an E. coli translation termination factor (RF1), in such a way that amyloid aggregation of the chimera resulted in ribosomes translating through a premature stop codon inserted in a reporter gene (Fig. 1D, bottom) (65). This was a *de novo* implementation in bacteria of an engineering concept from a yeast prion and enabled the development of a quick *in vivo* test for antiamyloidogenic compounds (65).

## SynBio GENERATION OF CYTOTOXIC AMYLOIDS IN BACTERIA AS DISEASE MODELS

Systems biology of human neurodegenerative amyloidoses revealed multiple cellular targets and pathways (66), which have been studied in detail in a number of cellular model systems, from yeast, worms, and flies to fish and mice, all of them sharing genes in common with humans (67). Although now in focus because of the emerging link between gut microbiota and neuroinflammation (35), bacteria had been dismissed as models of human amyloid diseases due to their remote genetic relatedness and to the lack of many complex signaling and trafficking pathways, as a consequence of the absence of both intracellular organelles and multicellularity. In addition, heterologous expression of proteins involved in human amyloid diseases usually is not detrimental to bacteria, yet bacterial proteomes undergo aggregation physiologically linked to stress and aging (68, 69). To test if the accumulated knowledge on human amyloidoses has actually led to a clear understanding of their essential molecular and cellular bases, it would be crucial to reconstruct an amyloid disease from its first principles, i.e., (i) converting a protein not related, even remotely, to any involved in disease into a cytotoxic amyloid and (ii) characterizing its propagation and cytotoxicity in a host to which amyloidosis is alien. Bacteria and SynBio have something to say on these topics.

RepA-WH1 is an amyloidogenic protein device with no homologues in the human proteome. Evolutionary pressure for efficient protein folding seems to have naturally counterselected any proteotoxic intracellular amyloidosis in bacteria. Therefore, to attempt the generation of a synthetic bacterial amyloidosis, RepA-WH1 was expressed in E. coli with a fluorescent protein fused to its C terminus, to visualize the chimera and replace WH2 (thus freeing WH1 of its role as a functional amyloid). This approach allowed tracing the generation of cytosolic aggregates detrimental to bacterial proliferation (70); testing the ability of these aggregates, once purified, to nucleate the growth of RepA-WH1 amyloid fibers *in vitro* (41, 70); and exploring the coaggregation dominancy of distinct mutant variants of the protein (71). Vertical segregation of protein aggregates in E. coli is usually asymmetric to guarantee that one of the two resulting cells inherits no damaged proteins (69). However, while tracking E. coli proliferation within microfluidic channels, aggregates of RepA-WH1 including the A31V hyperamyloidogenic mutation (see above) were segregated equally between the daughter cells, thus reducing the probability of getting bacteria “cured” of aggregates (47). Cells showed two distinct phenotypes: either multiple globular aggregates, which were very cytotoxic, leading to cell filamentation and death, or single elongated comet-shaped particles, which were mildly toxic and split on cell division (Fig. 1E, top). Both aggregate types were reactive to an amyloidotropic chemical probe, although this exhibited a higher affinity for the globular aggregates (47). Therefore, these two kinds of particles respond to distinct amyloid strains, i.e., a unique protein sequence adopting two or more alternative aggregate conformations, each one determining a different phenotype. While the elongated RepA-WH1 amyloid strain tends to spontaneously convert into the globular one, the bacterial DnaK (Hsp70) chaperone, but not ClpB (Hsp104), is able to revert such strain conversion in the bacterial cytosol, providing a convincing demonstration of the ability of an Hsp70 to detoxify amyloids *in vivo* (47). Interestingly, cell-free synthesis of RepA-WH1 inside lipid vesicles in the presence of chaperones reproduces aggregation as observed in bacteria (48). Finally, the expression under blue light illumination of a chimeric LOV2-WH1 optogenetic device in E. coli leads to the generation of oligomeric cytotoxic aggregates that hamper bacterial growth, underlining the central role of RepA-WH1 destabilization in triggering amyloid cytotoxicity (Fig. 1C) (63).

Differential transcriptomics, combined with functional assays and proteomic analysis of the proteins trapped with the RepA-WH1 aggregates, outlined the systemic routes of amyloid toxicity in E. coli (Fig. 1E, bottom) (72). As seen *in vitro* (58), RepA-WH1 makes holes in the internal bacterial membrane, thus draining the proton motive force, ATP synthesis, and any coupled transport (in particular, siderophore-mediated iron uptake). As a last resource, the expression of the alternative NdhII dehydrogenase, less efficient in respiration, generates reactive oxygen species (ROS) through auto-oxidation (72). A fraction of the E. coli proteome, including enzymes and transcription factors forming part of the bacterial response to oxidative and envelope stresses, specifically coaggregates with RepA-WH1, thus contributing to bacterial death (72). Overall, this picture resembles some of the core pathways described for mitochondria in amyloid neurodegeneration (73). Interestingly, the E. coli proteins that coaggregated with RepA-WH1 had a reduced number of predicted amyloidogenic stretches in their sequences, while those aggregating with a control RepA-WH1 variant, which had the amyloidogenic sequence truncated and formed noncytotoxic inclusion bodies (47), comprised multiple aggregation-prone segments (72). This observation is in line with the finding that the uptake of peptides including short aggregation-prone sequences with homology matches to a subset of the E. coli proteome resulted in the cytotoxic coaggregation of the targeted proteins (74). Beyond its potential in the generation of novel antibacterials, this concept has been recently extended to the design of synthetic antiviral amyloids (75).

To fulfill all the characteristics of a prion, a protein aggregate must also be horizontally transmissible, i.e., between donor and acceptor cells of distinct lineage, somehow matching the requirements for an infectious agent (2). This final condition is the hardest to match for a synthetic bacterial amyloid because the bacterial envelope (especially in diderm, Gram-negative bacteria) is a formidable barrier to influx permeability of large protein aggregates. Therefore, horizontal transmissibility of RepA-WH1 was recently probed in cultured mammalian cells (Fig. 1F) (76). First, the same RepA-WH1 variants that had been fused to a fluorescent protein and assayed in bacteria (see above) were expressed in the cytosol of murine cells: while those including the hyperamyloidogenic A31V mutation formed multiple cytotoxic amyloid aggregates, the wild-type (wt) protein remained soluble and the cells were viable. Then these viable cells were used as receptors in an experiment in which *in vitro*-assembled and fluorescence-labeled RepA-WH1 amyloid fibers were added to the culture (76). In an alternative approach, the murine cells experiencing the RepA-WH1(A31V) amyloidosis were cocultured, as donors of amyloid aggregates, with human cells stably expressing RepA-WH1(wt) fused to a distinct fluorescent protein. In both cases, RepA-WH1(A31V) amyloid particles were readily captured by the receptor cells, but unlike CsgA on sequence-unrelated mammalian proteins (36, 37, 77), intracellular aggregation occurred only in recipient cells expressing soluble RepA-WH1(wt), which could be molded into amyloid upon binding the internalized aggregates (76). It is interesting that vascin, a synthetic peptide from a nonamyloidogenic human protein (endothelial growth factor receptor 2 [VEGFR2]), like RepA-WH1, specifically seeds the aggregation of soluble full-length VEGFR2 in the recipient cells but not of any other intracellular protein (78). Finally, the proteomes of receptor cells that had been incubated with the *in vitro*-assembled or the *in vivo*-secreted RepA-WH1(A31V) amyloids were analyzed: the respiratory complexes in mitochondria, and also the protein trafficking and quality triage pathways, showed up in the cells expressing RepA-WH1(wt) but not in control cells expressing the fluorescent protein tags (76). These findings are in good agreement with the results on the toxicity exerted by RepA-WH1 in bacteria (72). Regarding mitochondria as preferential targets in amyloidoses (73), their dynamic membranes, the lack of a cell wall, and the existence of an efficient protein import machinery may explain their greater susceptibility to amyloid uptake compared to bacteria (79). It is noteworthy that the mere uptake of RepA-WH1 aggregates by the mammalian cells is not toxic: recipient cells are required to express the same protein in a soluble form. This constitutes a proof for the biosafety of the synthetic bacterial prion, in accordance with the absence of any significant match for RepA-WH1 in the human proteome. In summary, RepA-WH1 illustrates the potential of a “generic” synthetic bacterial amyloid engineered from first principles as a minimalist model for prion biology.

## FUTURE DIRECTIONS

To fulfill the current expectations on the use of amyloid nanomaterials in SynBio applications, achieving control of amyloid nucleation and growth and of any potential cytotoxic side effects is an essential goal. As addressed here, ligand-promoted amyloidogenesis can provide such fine-tuned levels of regulation and thus should be explored further. Conversely, engineering amyloid cytotoxicity in bacteria, coupled to horizontal gene transfer, might enable new ways to selectively eliminate either bacterial pathogens or specific bacteria from an engineered microbial consortia to program changes in strain composition in biotechnological processes, as proposed for light-modulated cell ablation by “optobiotics” (63). It might be interesting to test synthetic bacterial prion-like proteins, as generated by engineered microbiota, in the current animal models of human amyloidoses to assess their possible effect on disease onset and progression. Small bacterium-related mobile genetic elements encoding just a protein carrying a domain like RepA-WH1 have been isolated from bovine meat and dairies, and also from primary human tumors while the patients developed antibodies against the protein (80). So probably, there is still plenty of room to be explored for bacterial amyloids in human diseases.
